# Breast lesions classifications of mammographic images using a deep convolutional neural network-based approach

**DOI:** 10.1371/journal.pone.0263126

**Published:** 2022-01-27

**Authors:** Tariq Mahmood, Jianqiang Li, Yan Pei, Faheem Akhtar, Mujeeb Ur Rehman, Shahbaz Hassan Wasti

**Affiliations:** 1 Faculty of Information Technology, Beijing University of Technology, Beijing, China; 2 Division of Science and Technology, Department of Information Sciences, University of Education, Lahore, Pakistan; 3 Beijing Engineering Research Center for IoT Software and Systems, Beijing, China; 4 Computer Science Division, University of Aizu, Aizuwakamatsu, Fukushima, Japan; 5 Department of Computer Science, Sukkur IBA University, Sukkur, Pakistan; 6 Radiology Department, Continental Medical College and Hayat Memorial Teaching Hospital, Lahore, Pakistan; University Tunku Abdul Rahman, MALAYSIA

## Abstract

Breast cancer is one of the worst illnesses, with a higher fatality rate among women globally. Breast cancer detection needs accurate mammography interpretation and analysis, which is challenging for radiologists owing to the intricate anatomy of the breast and low image quality. Advances in deep learning-based models have significantly improved breast lesions’ detection, localization, risk assessment, and categorization. This study proposes a novel deep learning-based convolutional neural network (ConvNet) that significantly reduces human error in diagnosing breast malignancy tissues. Our methodology is most effective in eliciting task-specific features, as feature learning is coupled with classification tasks to achieve higher performance in automatically classifying the suspicious regions in mammograms as benign and malignant. To evaluate the model’s validity, 322 raw mammogram images from Mammographic Image Analysis Society (MIAS) and 580 from Private datasets were obtained to extract in-depth features, the intensity of information, and the high likelihood of malignancy. Both datasets are magnificently improved through preprocessing, synthetic data augmentation, and transfer learning techniques to attain the distinctive combination of breast tumors. The experimental findings indicate that the proposed approach achieved remarkable training accuracy of 0.98, test accuracy of 0.97, high sensitivity of 0.99, and an AUC of 0.99 in classifying breast masses on mammograms. The developed model achieved promising performance that helps the clinician in the speedy computation of mammography, breast masses diagnosis, treatment planning, and follow-up of disease progression. Moreover, it has the immense potential over retrospective approaches in consistency feature extraction and precise lesions classification.

## Introduction

Breast cancer is threatening malignancy and the leading cause of cancer-related mortality in women’s community, with an increased 6.6% to 6.9% mortality rate in the current year [1, 2]. This high death rate is primarily due to delayed malignancy detection. Breast cancer is curable if detected early, which increases the patient’s chances of survival [3]. Breast lesions are classified as calcification or mass based on their appearance, which may aid in the detection of breast malignancies. Masses are the most prevalent clinical sign of carcinomas that appear in mammograms as grey to white pixel intensity values. Timely detection of breast cancer masses is imperative for proper medication due to their modest size when patients exhibit no initial symptoms. Breast masses vary in intensity, distribution, shape (lobulated, irregular, round, oval) and boundary (spiculated, ill-defined, circumscribed) within the breast region, which increases the likelihood of misdiagnosis [4]. Breast cancer is categorized as malignant when tumors are irregularly shaped, have ambiguous edges, and blurred boundaries; on the other hand, benign masses are often dense, well-defined circumscribed, and roughly spherical. The hidden features nearby the masses area are crucial for breast cancer research [5]. As a result of the heterogeneity, morphological diversity, confusing boundaries, and varying cancerous cell sizes, doctors have difficulty recognizing malignant tumors, resulting in needless biopsies.

Medical imaging modalities, especially digital mammography, is a well-known and effective technique for timely screening, detecting, and measuring breast density [6]. On the other hand, mammograms are challenging to grasp and interpret due to their poor contrast, architectural complexity, and similarity of lesion intensity to normal tissue at the mass boundaries, making it difficult to recognize the breast masses. It is crucial to extract accurate mass characteristics from mammography images for mass recognition and analysis. Mammography analysis assists radiologists in detecting the location and size of breast masses, which is reassuring for potential treatment measures [7]. A radiologist usually performs mammography analysis manually, which is time-intensive, complicated, biased, and prone to significant expert variability. Misinterpreted mammograms lead patients to take hazardous measures, such as advised breast biopsies if malignant lesions are detected.

Reduced recall and biopsy rates are imperative for reducing patient stress and treatment costs while attaining optimal cancer detection measures based on individual needs [8]. Given these obstacles, the scientific community has made several efforts to enhance radiologists’ clinical performance by developing computer-aided diagnostic (CAD) systems to diagnose breast masses using mammography screening. The deep learning-based CAD methods are adversely affected by the inherent snags of mammography, such as noise and illumination. However, existing CAD methods are inefficient in reducing breast cancer mortality, and recall rates [9]. It is difficult to identify lesions from the neighboring healthy tissues, resulting in high false-(positive and negative) predictions. False-positive prediction needs more intensive treatments such as re-screening and biopsies, incurring excessive anxiety and pain [10]. Deep learning-based techniques for detecting and classifying high-risk lesions have been used to reduce human error rates in predicting breast lesions [11]. Developing robust deep learning-based approaches, especially the convolutional neural networks (CNN), can relieve the doctor’s pressure and improve automated mass detection, mass localization, feature learning, and classification [12]. Despite training challenges owing to a lack of labeled images, CNN preserves mammography’s spatial integrity, such as how pixels are linked to form a distinct feature. Moreover, deep learning-based methods still face significant problems acquiring massive annotated training images and ensuring generalizability across cohorts, devices procurement, and modalities.

Recent advances in deep convolutional neural network (DCNN) models have enabled the automated detection of breast anomalies without the need for extensive training data by effectively modifying different parameters [13]. Many DCNN architectures are available, including VGGNet [14], Inception [15], ResNet [16], and EfficientNet [17], each with its own distinct design that is tailored for a particular grading task. DCNN architectures are coupled with transfer learning (TL) paradigms to recognize suspect breast areas accurately in mammography images, enhancing radiologists’ screening abilities. TL is a popular deep learning approach for detecting, learning features from lower layers, and classifying breast masses with fine-tuned hyperparameters [18]. Model fine-tuning through TL is much more affordable and efficient than starting with dynamically initialized weights. Moreover, the hybrid categorization study outperformed both learned and hand-crafted models [19].

This study aimed to examine the gap in predicting breast masses’ region of interest (ROI) on mammograms using computer vision algorithms. However, this research proposed a convolutional neural network that achieves state-of-the-art performance in classifying the ROI of breast masses, enabling physicians to detect even the most minor breast masses early. Each image in the MIAS and Private datasets is preprocessed to eliminate noise and improve image quality by proposing different enhancement approaches. Afterward, the ROI of benign and malignant classes is cropped according to each cancerous area’s specific coordinate, and small patches are extracted from the cropped ROI. Our approach efficiently extracts low-level features, reduces variability and generalization inaccuracy, and improves lesion classification using cropped image patches. This research transforms unbalanced data and significantly reduces computing time and false-(positive and negative) predictions. Convolution filters are employed to obtain spatial information of a broader region while retaining computational efficiency. Besides, the transfer learning paradigm is proposed to enhance the standard pre-trained methods by modifying the final layer to classify ROIs of breast masses accurately. We demonstrate our technique’s effectiveness by comparing it to other state-of-the-art approaches using the MIAS dataset as the gold standard. The proposed model and five standard CNNs pre-trained architecture are evaluated with different evaluation metrics such as AUC, sensitivity, recall, precision, and accuracy. As a result, the developed model aims to help experts in treatment planning and decision-making based on cancer’s initial symptoms to detect and classify suspicious areas from mammography.

The rest of the proposed work is structured as follows. A description of the latest deep learning diagnosing and grading approaches are presented in Section. Section describes the technical strategies for breast mass detection and classification, datasets, and mammogram image preprocessing. Section depicts the investigation of proposed architecture’s performance and experimental findings. In Section the details of the detection and classification findings are carried out and discussed through various evaluation parameters. Finally, Section summarises the important research work’s outcomes and future directions.

## Related work

A mammography-based computer-aided diagnosis system enables timely breast cancer detection, diagnosis, and medication. CAD systems improve the effectiveness and performance of breast mass diagnosing [20]. Different medical imaging modalities substantially lower the rate of false-positive prognosis to improve the predictive ability of breast mass. Due to the heterogeneity of breast density and mammography’s low contrast, feature selection and manual feature extrication are computationally challenging and time-intensive [21]. However, deep learning-based CNN algorithms have improved breast mass detection and classification by learning features from raw breast images.

The research community achieved remarkable improvement in predicting breast cancer based on Deep CNN by minimizing the drawbacks of standard mass detection approaches. Guan et al. [22] develop an approach for recognizing and localizing breast tumors in digital mammograms based on regions of interest paired with a DCNN. The suggested method employs asymmetry information from a pair of breasts from the same individual to improve detection accuracy. Shu et al. [23] constructed a deep CNN classification model using two pooling structures rather than more conventional pooling approaches. The proposed technique entails two stages: feature extraction for feature learning and pooling structure for segmenting mammograms into subregions with a high risk of malignancy using the retrieved features. The model attained 0.922 accuracy with a 0.924 AUC on INbreast and 0.76 accuracy with an 0.82 AUC on the CBIS-DDSM databases. Samala et al. [24] devised a deep learning strategy combined with transfer learning to extract and classify the in-depth feature into cancerous and non-cancerous lesions on mammography. Breast mass classification was employed to assess generalization error, and the findings exceeded analytically derived features. Lee et al. [25] develop a fully automatic deep learning-based method for segmenting and classifying the dense areas in mammography. A full-field digital screening mammography dataset was used to assess the model’s efficacy.

Ragab et al. [26] developed a deep learning-based algorithm for feature learning and classification to assist clinicians in detecting breast anomalies in mammograms. The proposed approach retrieved in-depth training features and tested the support vector machine classifier applying multiple kernel functions. The experiments were performed using the MIAS dataset and obtained an accuracy of 0.97, much higher than earlier methodologies. Tan et al. [27] designed a deep learning-based approach for detecting and classifying breast lesions as benign or malignant. The research speeds up the diagnostic process by assisting doctors in diagnosing breast masses and yields a higher accuracy of 0.82 in mass detection than previous approaches. Hadash et al. [28] represent a CNN-based approach for detecting, localizing, and classifying abnormal breast lesions. The design model was validated on digital database for screening mammography (DDSM) dataset, yielding an accuracy of 0.91, sensitivity of 0.94, and AUC of 0.92. Choukroun et al. [29] proposed a multiple instances learning (MIL) technique for the automated diagnosis and grading of breast anomalies without the stipulation of annotated data. The technique’s distinguishing feature identifies discerning regions across the mammography image, overcoming classification inaccuracies. Omonigho et al. [30] used the DCNN model to classify the mammographic images. The methodology aims to extract features and segment ROI using threshold approaches by modifying the last layer of the DCNN with the SVM model. The results demonstrated that the model’s accuracy improved. Daniel et al. [31] applied CNN fused with TL to categorize the pre-segmented breast masses as malignant or non-cancerous. Data augmentation approaches were used to reduce the training sample’s deficiency, resulting in 0.92 accuracy.

Accurate mammography classification by deep learning model has significant benefits, including reducing annotation, improved use of contextual information, lower call-back rates, and unnecessary tests without sacrificing the model’s sensitivity. Despite traditional research for malignancy diagnosis having particular challenges, the following are the most significant:

- The scarcity of breast images poses a significant barrier in attaining an efficient classifying accuracy. Acquiring breast images from a particular vendor is tedious and expensive for training and validating breast mass classification techniques.
- Unbalanced data in training datasets are frequent, resulting in poor model performance on small datasets.
- Hence, deep learning-based models are adversely affected by the inherent snags of mammography, such as noise and illumination, so a technique for noise reduction is required.

Presently, the concept of automatic detection and classification of breast lesions is gaining momentum, and radiologists continue to face gaps and challenges [32]. Existing studies expose that the CAD systems are ineffective in improving mammography diagnostic accuracy due to a lack of training data. Acquiring training data comprising breast cancer-related features and anomalies is crucial for conducting realistic analysis. Although benchmark datasets are widely obtainable, it requires considerable effort to erect a live medical dataset and image it in a laboratory [33]. Thus, deep learning methods are widely used for automated detection, requiring massive training data encompassing all features and variations correlated with breast cancer. Few studies use large-image datasets (ImageNet) to train the CNNs classifiers by fine-tuning the hyperparameter with transfer learning. Consequently, the proposed method employed data augmentation and transfer learning approaches to overcome the flaws above and obtain accurate breast mass predictions.

## Materials and methodology

**Fig 1:**
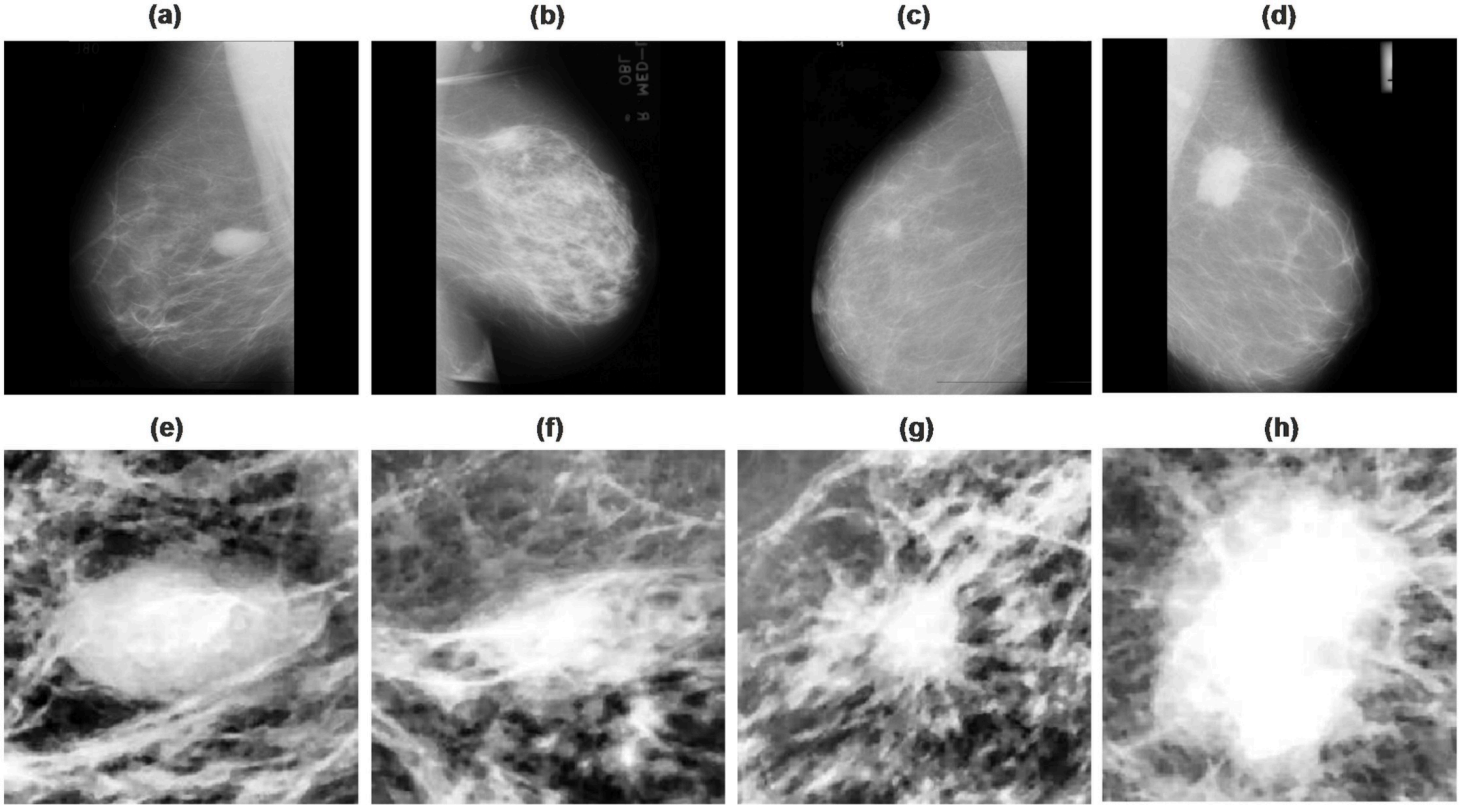
Few samples of digitized mammography images collected from the MIAS dataset. (a-b) are benign tumors, (c-d) malignant tumors, (e-f) sample of extracted ROIs of benign tumors, (g-h) extracted ROIs of malignant tumors.

Breast cancer is caused by rapidly developing aberrant cells threatening women’s health. The timely detection increases the patient’s chance of survival. Breast mass is a marker that enables radiologists to segment benign and malignant tissue during diagnosis [34]. The Fig 1 depicts the malignant and benign breast tumors in a mammography image with cropped suspicious areas. This research requires immense mammography images labeled as benign or malignant collected from Private and MIAS datasets. Expert radiologists manually labeled the mammography images in the Private dataset as benign or malignant, verified and diagnosed with ground truth data. The proposed study was approved by the IRB of Continental Medical College & Hayat Memorial Teaching Hospital, and the requirement to obtain informed consent was waived due to privacy and ethical concerns.

The proposed framework is based on four stages for predicting breast lesions: mammography preprocessing and data enhancement, suspicious ROI detection and segmentation, feature learning, and mass classification. The raw mammography images affected by the inherent noise and illumination snags need more clarity, denoising, and normalization. First, each image in the MIAS and Private datasets is preprocessed to eliminate noise and improve image quality by proposing different image filters and enhancement approaches [35]. Afterward, the region of interest of benign and malignant classes is cropped according to each cancerous area’s, and small patches are extracted from the cropped ROI [36]. Our approach efficiently extracts low-level features, reduces variability and generalization inaccuracy. This research transforms unbalanced data and significantly reduces computing time and false-(positive and negative) predictions. Furthermore, the transfer learning paradigm is proposed to enhance the efficiency of five pre-trained methods on modifying the final layer. The six proposed models are evaluated on the MIAS and Private datasets with different evaluation metrics: AUC, sensitivity, recall, precision, and accuracy. The proposed work’s main contribution may be characterized as follows:

- To develop a novel approach for automating diagnosis, localization, and classification of breast cancer masses based on the CNN architecture.
- To evaluate the prediction ability of the transfer learning paradigms in mammography by comparing five pre-trained algorithms fine-tuned over two distinct datasets.
- To identify and crop ROIs for breast masses using a brute force global thresholding technique combined with a morphological operation to overcome mammography’s bootless parts.
- To predict the most suspicious areas using a region-based max-pooling framework with the small-sizes kernel work to reduce overfitting complexities.
- To assess the proposed models’ performance using a novel manually labeled Private dataset and compare it to other state-of-the-art schemes employing the MIAS dataset as the gold standard.

### Datasets

Collecting real-time medical images for research purposes is exceptionally problematic due to confidentiality concerns. Each image used in this research was obtained from publicly MIAS and local Private datasets. The detailed description of both databases as:

MIAS (Mammogram Image Analysis Society) [37] a public dataset consists of 322 digital mammographic images of 161 cases, including different view images of both breast and precise annotations. It contains 64 images as benign cases, 52 as malignant, and 207 as normal, verified, and diagnosed with experts’ ground truth data. Each image is a gray-scale with a 1024 × 1024 pixel size with a 200 micron resolution stored in (*PGM*) format, including distinct types of lesions (calcification, mass, asymmetry, and distortions) and pathologic ground truth about prognosis. Each abnormal sample is labeled with the degree of the aberration, and the number of benign and malignant cells is not normalized. We distinguished between normal and abnormal, as most previous research on the MIAS dataset has performed. Additionally, the ROI was cropped applying coordinates of the centre and the radius of the anomaly given by the dataset. In the current study, only BIRADS (Breast-Imaging Reporting and Data-System) annotations as benign (∈ 2, 3) and malignant (∈ 4, 5, 6) are used.

The Private dataset was obtained with approval **(Approval number: MG59308)** of the Institutional Review Board (IRB) of Continental Medical College and Hayat Memorial Teaching Hospital, Lahore, Pakistan [38] for this research. The novel dataset consisting of full digital mammograms images is available for research use after request. The Institution Board Committee / IRB of Continental Medical College and Hayat Memorial Teaching Hospital approved the proposed study. The hospital obtained the consent for experimentation purposes without disclosing patients’ personal information due to privacy and ethical concerns. The radiologist team consisted of two senior radiologists with eighteen years of expertise dealing with diagnosing suspicious regions such as masses and calcification in mammograms. The professional radiologist manually labeled the original mammography images into benign and malignant classes based on the initial screening and diagnosis reports of the patients created by the mammography screening machine. These annotations served as the basis for the creation of the ground truth labels. The dataset was first analyzed using the suggested techniques for increasing the sensitivity of breast mass prediction through a fully automated CAD system. The Private dataset contains 482 positive images as a malignant tumor and 98 negative images as a benign tumor with a total of 580 mammographic images. The patients ranged in age from 30 to 78 years, with a mean of 49.05 years. Each image is a gray-scale in Digital Imaging and Communications in Medicine (DICOM) format with both standard bilateral MLO (mediolateral-oblique) and CC (craniocaudal). Every breast has two projections (left and right), one for each instance. The spatial resolution is determined by the mammography equipment employed, and the aforementioned mammogram image has a spatial resolution of 3328 × 4096 pixels. Each mammography was scanned using a laser film digitizer with 100*m* spatial resolution and 12*bits* contrast sensitivity for feature detection. The focal length of each CC and MLO view of the mammogram was 35 mm, with a pixel resolution of 85*μm* along the horizontal and vertical axes. All mammograms were collected from distinct patients to mitigate the probability of overfitting in the classification method created by the similarity of mammograms from a single patient.

The datasets were randomly split into training, testing, and validation sets in the proportions of 60%, 20%, and 20%, respectively. Both datasets were enhanced using appropriate augmentation methods to reduce overfitting. It comprised 5568 augmented mammography images split into training, testing, and validation sets to maintain the same cancer case rate. We employed 3341 mammography images (60% of the dataset) in the training set, 1114 (20% of the dataset) in the test set, and 1113 (20% of the dataset) in the validation set to assess the proposed model’s accuracy.

### Mammogram preprocessing

In collected mammograms, a significant number of anomalies are misinterpreted due to poor-quality images with artifacts, pectoral tissue, low visibility, and interference with noise, which impair the image’s clarity, resulting in a high false-positive rate. Preprocessing of images improves computational quality, image smoothing, and noise mitigation [39]. We employ noise reduction methods such as Median, Gaussian, and Bilateral filtering to eliminate unsharp masking and efficiently denoising images caused by gaussian, salts, and pepper, and speckle noise while retaining sharp edges.

Afterward, image enhancement approaches increase the image’s visual characteristics, such as margins, edges, and contrasts, and decrease imperfections. This study proposes the contrast limited adaptive histogram equalization (CLAHE) method to enhance the overall quality of mammography images [35]. The global threshold method such as Otsu’s thresholding is used to crop the mass region and remove the black spaces in mammography, followed by standard morphological techniques [36]. As a result, the cropped masses’ ROI contains breast tissue solely, without pectoral tissue, label, or background noise. Furthermore, data normalization is utilized to reduce visual variability within a dataset and enable fast CNN learning [40]. Finally, without the intervention of an expert, the in-depth feature of suspicious regions is retrieved to detect breast cancer masses and calcification.

#### Data augmentation

**Fig 2:**
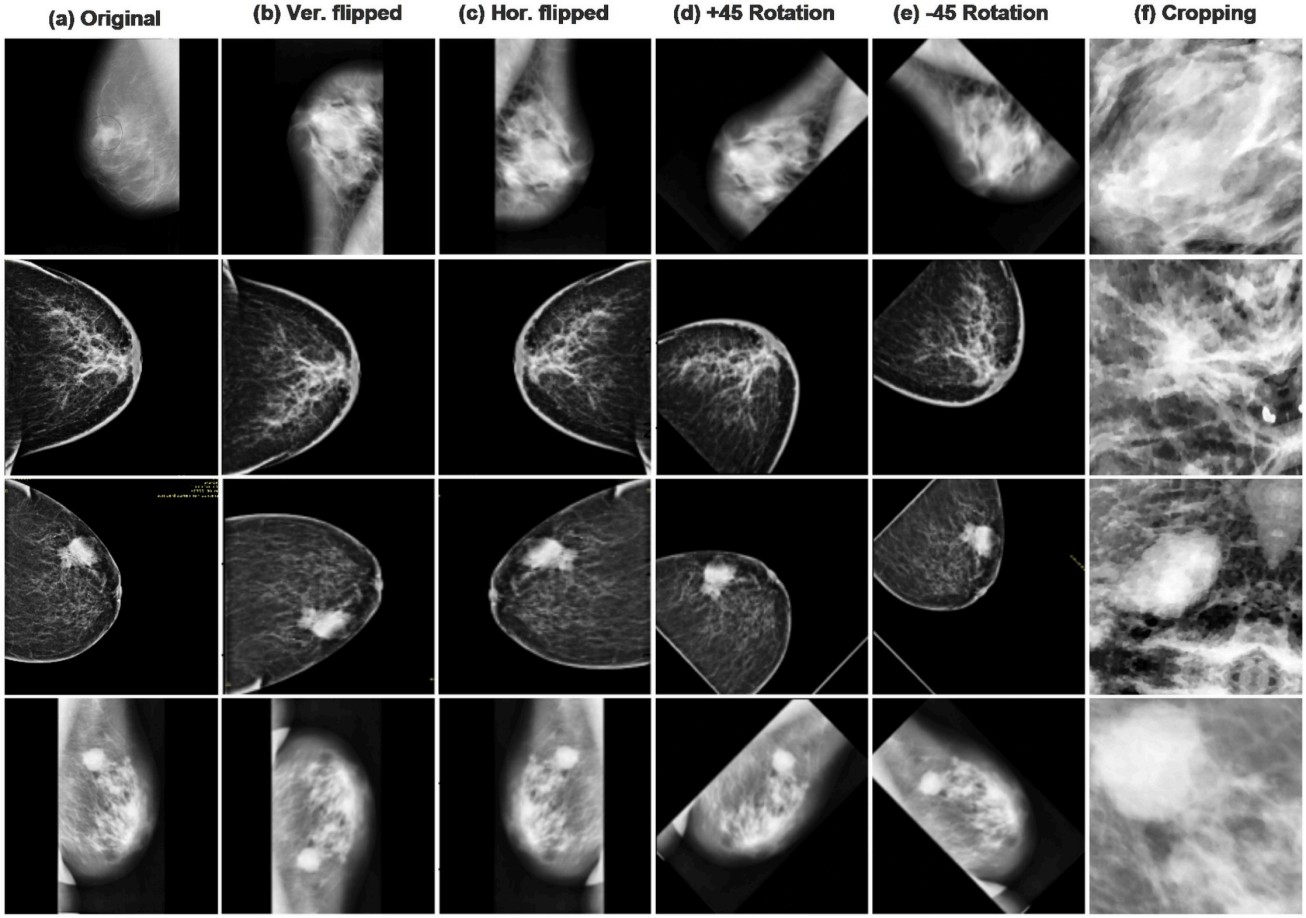
Randomly selected samples of mammographic images used after preprocessing and enhancement, starting with original and augmented images (a) Original raw mammogram image with breast masses, (b) Vertical flipped, (c) Horizontally flipped, (d) Rotation +45, (e) Rotation -45, and (f) Cropping.

Data augmentation is a remarkable approach for mitigating overfitting, boosting the model’s generalization, and improving efficiency. Overfitting in CNN-based architecture occurs when models learn too many features from training data yet do not generalize well enough to make accurate predictions about unseen future data. As a result, the trained model does not perform well on testing data. This is often the case when the training data set’s quantity is insufficient compared to the number of model parameters to be trained. Thus, this research artificially inflates the datasets eight times by using several data augmentation methods as shown in Table 1. The enlarged dataset is created by executing geometric transforms on the small dataset, such as scaling, flipping, rotating, translations, and cropping [41]. Fig 2 shows the discrete data augmentation approaches used to inflate images in both datasets. Images are rotated between (+*pm*45 degrees) to mitigate the effect of overfitting across all training epochs. Keras’ ImageDataGenerator library is used to build batches of tensor image data with real-time data enhancement [42]. Hence, this study creates the 5568 mammogram images that contain 1296 benign and 4272 malignant.

**Table 1:** Different ques of augmenting data with invariance parameters.

Sr	Augmentation methods	Invariance Parameters
1	Sharpen (lightness value)	0.5, 1, 1.5, 2
2	Emboss (strength value)	0.5, 1, 1.5, 2
3	Gaussian Blur (Sigma value)	0.25, 0.5, 1, 2
4	Rotation	45°, 90°, 135°, 180°
5	Edges Detection (alpha value)	0.25, 0.5, 0.75, 1.0
6	Skew (Tilt)	Forward, Backward, Left, Right
7	Flipping	Top, Bottom, Left side, Right side
8	Shear (axis & value)	X-axis 10°and Y-axis 10°

#### Cropping

The mammogram images are often large in size, and only 30% area contains the breast tissue portion, whereas the background and pectoral muscle occupy the rest. In the non-breast area, tissue density is strongly linked, which may influence subsequent mammography analysis [43]. Therefore, instead of screening the whole mammogram, the suspicious region containing the intensity changes is processed Cropping inflates datasets by integrating the spatial dimensions of mammography images without affecting their labels [44]. Breast tumor intensities are reduced initially by using a spatial interpolation method to extract local characteristics. Fig 2f illustrates the cropped images.

Each mammogram’s abnormal areas are labeled with the center coordinate and estimated radius. The ROIs, including the annotated areas, are retrieved in this study to get more detailed global morphological information for each lesions region. This study used the global thresholding method such as Otsu’s thresholding to crop the masses area and remove black spaces in mammography after morphological operations. Otsu’s approach determines a threshold value that efficiently discriminates between low- and high-density areas. Following a similar approach, we cropped the patches of ROI masses to a size of 224 × 224 pixels in both datasets. Consequently, the cropped masses’ ROI contains breast tissue solely, without pectoral tissue, label, or background noise.

#### Patches extractions and normalization

The sliding window technique was adopted to conduct coarse localization of suspicious areas. The patches are generated from the centroids of suspicious regions in a mammogram using a partially overlapped window size 222 × 224 This technique enables patch extraction based on the size and location of the suspicious region. Moreover, we also applied the horizontal reflection approach of data augmentation to enhance dataset size [45]. Each patch is labeled based on whether or not it includes a lesion. Patches of different classes were maintained separately for feature extraction and to ensure model accuracy throughout training [43]. Furthermore, low-level mammogram features such as shape, texture, area orientation, perimeter, and intensity for mammogram patch detection are commonly used. The cropped ROIs were downsized and processed into the spatial resolution of 222 × 224 as an input to suit the standard/expected input sizes for all adopted models. Larger window sizes and downsizing were done to speed up processing despite maintaining a high degree of resolution. Furthermore, Contrast normalization methods are used to normalize the patches, in which the impact of intensities is subtracted from the corresponding pixel.

### The experimental architecture of CNN-based model

**Fig 3:**
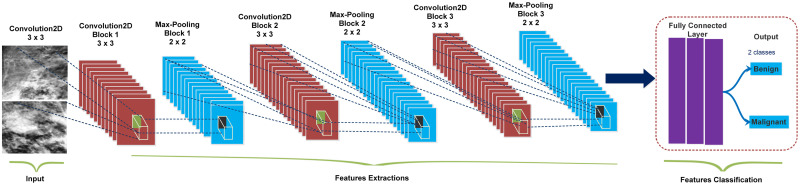
The layered framework of the designed CNN-based model (ConvNet) consists of 3 × 3 Con_2D convolutional layers, followed by the max-pooling layers and softmax classifier for classifying mammography abnormalities as malignant or benign.

**Fig 4:**
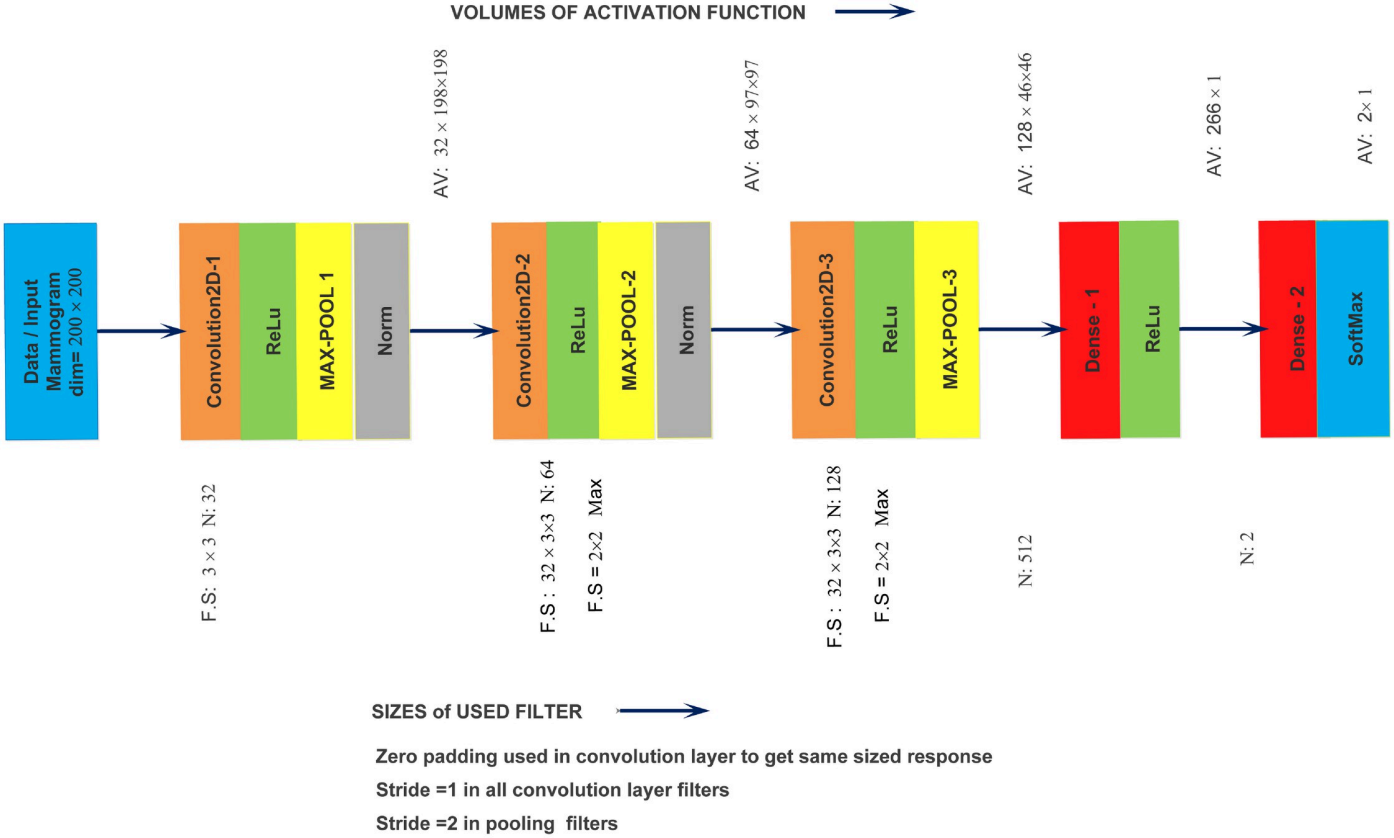
The implementation of designed ConvNet architecture containing convolution2D, ReLu, max-pooling, batch normalization and softmax layer for the classification of breast masses.

Deep CNNs have achieved tremendous strides in breast lesions diagnosing, segmentation, feature learning, and classification. The deep neural network uses attributable sparsely connected kernels to determine the density of medical images. The CNN-based model comprises three types of layers: the number of convolution layers (input), pooling (hidden), and one or more dense or fully connected layers (output). Fig 3 illustrates a CNN-based architecture with multiple convolutions, pooling, and dense layers. The proposed lightweight ConvNet model contains twelve weighted layers, out of which four convolution layers for in-depth feature learning integrated with rectified linear unit activation. Convolutional layers progressively reduce the dimensional resolution of mammograms while increasing the depth of their feature maps. Convolution was performed using a (3 × 3) kernel filter, the stride of (1 × 1), and the same padding. Afterwards, the max-pooling layers obtain vector representation from extracted in-depth features to perform data reduction. Max-pooling was performed using stride (2 × 2) and kernel filter (2 × 2) to get the best results. A dense and softmax activation layers are used for tumor classification by reducing the over-fitting problem. The normalization layer is added after the convolution layer to ensure that the input data distribution for each layer is more consistent. An activation function is used after the pooling layers to minimize feature dimension computations, speed up convergence, and enhance the network’s nonlinearity. However, the first convolution layer, on the other hand, is followed by a max-pooling layer, and the subsequent three layers follow the same pattern, as shown in Fig 4.

We retained certain features of the original model, such as the convolutional and max-pooling layers, to efficiently obtain information. In the proposed architecture, feature extractor is denoted as *e*_*f*_. Input image *x*, *z* = *e*_*f*_(*x*|*ϕ*), here *zϵR*^*w*×*h*×*c*^ represent its feature mapping, *w* the row, *h* the column of *z*, *c* channel dimension, and *ϕ* is network parameter. Each layer in the CNN-based model’s feature extraction stage aggregates data from the preceding layer and sends it on as input to the subsequent layer until the feature classification stage delivers the final predicted results.

The *p*^−1*th*^ layer’s output is used as an input to the *n*^*th*^ layer, which then passes all inputs via kernels set followed by the nonlinear activation function. Eq 1 depicts the the convolution operation’s output matrix.

$$\begin{array}{c} c_{j}^{p}=e_{f}(\sum_{i}^{p-1}x\times k_{i.j}^{p}+b_{j}^{p}) \end{array}$$ (1)

Where $x_{i}^{p^{-i}}$ is as an inputs from *p*^−1*th*^ layer, $K_{i,j}^{p}$ is the kernels matrix of 1^st^ layer, and $b_{s}^{p}$ depicts the biases add to the 1^st^ layer. $c_{j}^{p}$ output matrix calculated by non-linear activation function *f* implemented on every element. The activation functions most commonly used in convolution layer are sigmoid and hyperbolic function as illustrated in Eq 2.

$$\begin{array}{c} e_{f}\left(x\right)=\frac{1}{1+exp(-x)}\;\;e_{f}\left(x\right)=tanh\left(x\right) \end{array}$$ (2)

The convolutional layer’s dimensionality is reduced using the max-pooling layer. During model training, the dropout technique is utilized to deactivate neurons dynamically, with an average dropout ratio of between 0.3 and 0.6. Max-pooling takes a feature map as input and returns a vector representation. The max-pooling method as feature extractor is denoted by *v* = *p*_*f*_(*z*|*β*), where *β* denotes the parameter, *p*_*f*_ max-pooling mapping, and *v* weight vector. The features obtained are combined to compute the final feature vector fed into the classification layer to predict malignancy likelihood in a mammogram. Each output dimension contributes to the optimal structural size by downsampling all inputs with 2 × 2 sized kernels. Eq 3 represents the pooling function.

$$\begin{array}{c} c_{j}^{p}=p_{f}(x_{i}^{p-1}) \end{array}$$ (3)

The current approach contains *w* × *h* × *c* for feature mapping and *w* × *h* for patches of dataset images. The *v*_*ij*_ vector corresponding to the patch *Q*_*ij*_ of input image *x*_*n*_. Here *i* *ϵ*{1, 2, 3, ‥*w*} and *j* *ϵ*{1, 2, 3, ‥*h*} presents the dimensionality of feature map row and feature map column respectively. If the whole the mammography screening is selected to determine the malignancy possibility, the pooling method can be formulated as in Eq 4.

$$\begin{array}{c} \left\{ \begin{array}{c} v=\frac{1}{w\times h}\sum_{i=1}^{w}\sum_{j=1}^{w}v_{ij}\\ p=\Theta (\frac{1}{w\times h}\sum_{i=1}^{w}\sum_{j=1}^{w}wv_{ij}=b) \end{array}\right. \end{array}$$ (4)

The last layer of the ConvNet model is used to determine the possibility of malignant or benign lesions, taking weight vector *v* as input. The suitable feature mapping can achieve the maximal befitting features of mammographic images for precise classification in terms of improved accuracy. The malignant lesions predication can be computed as declared in Eq 5.

$$\begin{array}{c} p=\Theta (w.v+b) \end{array}$$ (5)

here Θ is represents the active function, *w* prediction layer, and *b* bias. The loss used in the current architecture is illustrated as in Eq 6.

$$\begin{array}{c} L=-\frac{1}{N}\sum_{n=1}^{N}\left(w_{n}\left[t_{n}logp_{n}+\left(1-t_{n}\right)log\left(1-p_{n}\right)\right]\right)+\frac{\lambda }{2}{\left||\theta |\right|}^{2} \end{array}$$ (6)

where *t*_*n*_ *ϵ*{0, 1}, is the true label 0 represents the normal/benign, 1 the malignant label of malignancy for mammography image *x*_*n*_, *w*_*n*_ is a manual re-scaling weight assigned to the loss, λ denotes regularizer that regulates the complexity of the model, and *θ* deep network parameter. The architecture’s softmax layer contains a neuron output based on predicted classes with confidence in the prediction results. The final layer’s output of CNN is fed into a dense layer to obtain the projected classification results using softmax. The softmax operation for *i*^*th*^ class of input image *x*, wight vector *v*, and linear function *k* can be declared in Eq 7.

$$\begin{array}{c} S\left(y=i|x\right)=\frac{exp(x^{t}v_{i})}{\sum_{k=1}^{k}exp\left(x^{t}v_{k}\right)} \end{array}$$ (7)

The proposed model’s goal is to accurately detect lesions during testing and training by using segmented ROI patches from mammography images. Thus, the algorithm exhibits comparable prediction performance.

### Striding for feature reducing

Convolution’s output pattern is regulated by different factors, including the filter count and stride size. The stride parameter specifies the magnitude of the filter’s modification of the input data. Traditionally, a pooling technique is used to overcome the feature map’s resolution; however, the proposed method subsamples using a more extensive stride convolution process. Higher stride aids in the development of a standard CNN classifier by reducing dimensionality. The feature reduction using CNN minimizes the cognitive wight by subsampling the feature vector generated in the preceding convolutional layer. Forward propagation starts with an input image, and subsequent convolution layers gain new characteristics by analyzing the image’s filters.

### Transfer learning

Transfer learning offers a methodology for leveraging existing and trained architectures to tackle a new task domain in a relevant field. Training of CNN-based model is challenging with a limited amount of mammogram/medical images, which has been overcome using transfer learning and data augmentation techniques [46]. The demand for transfer learning in the medical realm occurs due to scarcity, high cost, and the unavailability of public datasets that are time-consuming to collect and label from professional radiologists. Moreover, training a deep learning-based model requires a significant amount of computing and memory resources. This study reuses CNNs such as VGGNet19, InceptionV3, ResNet152V2, InceptionResNetV2 [47], and EfficientNetB5 pre-trained over ImageNet to fine-tuned over mammogram images to detect and classify the breast masses. The proposed pre-trained architectures are fine-tuned by modifying the final fully-connected layers with new layers to discriminate the breast masses between two classes rather than 1000 [48]. Fine-tuning enables easy training and mitigates over-fitting.

TL improves the proposed architecture’s performance by extracting in-depth features from image data and applying them to the domain-specific and smaller dataset [21]. The pre-trained models are enhanced to develop large-scale feature vectors to extract low-level features for breast mass classification. The experimental findings reveal that the highest training accuracy rates are obtained 0.98 for ConvNet+Softmax compared to 0.93 for VGGNet19+Softmax, 0.82 for InceptionV3+Softmax, 0.82 for ResNet152V2+Softmax, 0.78 for InceptionResNetV2+Softmax, and 0.70 for EfficientNetB5+Softmax.

### Performance measures

The proposed models accurately classified the detected breast mass’s ROIs into malignant and benign classes and improved overall model performance. We examine the robustness of the model using different evaluation parameters on the Private and public MIAS datasets. We employ the stratified k-fold cross-validation approach (5-fold cross-validation) to assess the efficiency of the proposed ConvNet and pre-trained models in terms of true-positive and false-positive rates after adjusting model hyperparameters. Accuracy, sensitivity, F-score, precision, and area under the curve are calculated using the following equations.

The **accuracy** assessment parameter is calculated using Eq 8.

$$\begin{array}{c} ACC=\frac{1}{N}\sum_{i=1}^{N}\frac{TP+TN}{\left(TP+FN)+(FP+TN\right)} \end{array}$$ (8)

Here, true-positive (TP) results represent the lesions diagnosed as malignant, true-negative (TN) accurately diagnosed as benign. The false-positive (FP), benign lesion misinterprets as malignant, and false-negative (FN) malignancy misinterprets as benign. N represents the number of test times.

**Area under the curve**: AUC measures a classifier’s ability to discriminate between benign and malignant mammograms. The suggested method’s receiver operating characteristic (ROC) values were commutated to one. The ROC curve depicts the true-positive rate (TPR) as a function of the false-positive rate (FPR). The TPR and FPR are given in Eq 9 and are referred to as sensitivity (recall).

$$\begin{array}{c} Sensitivity=\frac{TP}{TP+FN}\times 100 \end{array}$$ (9)

The **precision** of a prediction is defined as the ratio of precisely predicted positive observations to all correctly predicted positive observations. The low FPR is associated with high accuracy. Using Eq 10, we can get the precision.

$$\begin{array}{c} Precision=\frac{TP}{TP+FP}\times 100 \end{array}$$ (10)

**F-Score** is the cumulative mean of precision and recall as illustrated in Eq 11, which is used independently to determine the correctness of test datasets.

$$\begin{array}{c} F-Score=2\times \frac{\frac{TP}{TP+FP}\times \frac{TP}{TP+FN}}{\frac{TP}{TP+FP}+\frac{TP}{TP+FN}} \end{array}$$ (11)

## Results and analysis

The proposed method was designed based on scientific methodology to predict breast masses using mammography images. Each image of both datasets is preprocessed to eliminate noise and improve image quality using different enhancement approaches. To analyse both datasets, tests were carried out on the proposed ConvNet and five pre-trained DCNN architectures. The findings show that our suggested technique outperforms DCNN algorithms and previous research.

### Experimental configuration

All experimental work are evaluated using Intel(R) Core (TM) *i*7 − 7700, CPU 2.80*GHz* and 2.80*GHz*, 16*GB* memory, NVIDIA GTX 1050*Ti* graphics card. The computation time for training and testing each CNNs model was 39 minutes on the Private dataset and 43 minutes on the MIAS dataset. Moreover, using Keras’ ImageDataGenerator library and Python using OpenCV, this study creates batches of mammography with real-time data augmentation and preprocessing. We consider the optimal hyperparameters which include batch size, learning rate, and optimization function, as described in Table 2.

**Table 2:** Hyperparameters configurations of proposed architecture.

Parameters	Values
Batch Size	32
Learning Rate	0.001
Epochs	90
Steps per epochs	80
Weight Decay	0.00005
Dropout	0.5
Momentum	0.9
Activation function	Softmax
Optimization function	Adam
Training Split	0.6
Testing Split	0.2

### Comparison between proposed method with other DCNN pre-trained models

Each experiment assessed the performance of the proposed model and compared it to five well-known pre-trained DCNN models. Each proposed model was evaluated in a similar training and testing environment. It could be observed in Table 3, the performance of our proposed model was much better than VGGNet19, InceptionV3, ResNet152V2, InceptionResNetV2, and EfficientNetB5. For both the Private and MIAS datasets, the efficacy of experimental results was assessed using a 5-fold cross-validation test. We adjusted the final layers of DCNN algorithms using transfer learning paradigm to extract features layer by layer and obtain locally and globally features through low/high-level learning. The efficiency of the model is increased by iterating various hyperparameter settings during model training.

**Table 3:** Performance comparison of proposed model with pre-trained models on Private dataset.

Algorithms	Precision	Sensitivity	F-Score	Accuracy	AUC
VGGNet19	0.93	0.92	0.91	0.93	0.88
InceptionV3	0.84	0.84	0.82	0.82	0.92
ResNet152V2	0.84	0.84	0.87	0.82	0.92
InceptionResNetV2	0.79	0.77	0.81	0.78	0.88
EfficientNetB5	0.71	0.61	0.71	0.70	0.79
Proposed Model	0.98	0.99	0.98	0.98	0.99

The performance of the models is evaluated using a training and testing dataset consisting of 3341 and 1114 mammography images, respectively. Initially, each proposed architecture is trained using the Adam optimizer with a learning rate of 0.001 and momentum constant of 0.9 [49]. The algorithm is trained over a period of 90 epochs at a rate of 80 steps per epoch, with a batch size of 32, and *L*2-Regularization. As training time to stop the activations, we include the dropout [50] with a likelihood of *p* = 0.5. MSRA [51] wight filters parameter used with 0.00005 weight decay to chastise large-weight and prioritize smaller. The MSRA technique was applied to initialize each layer’s weights.

**Fig 5:**
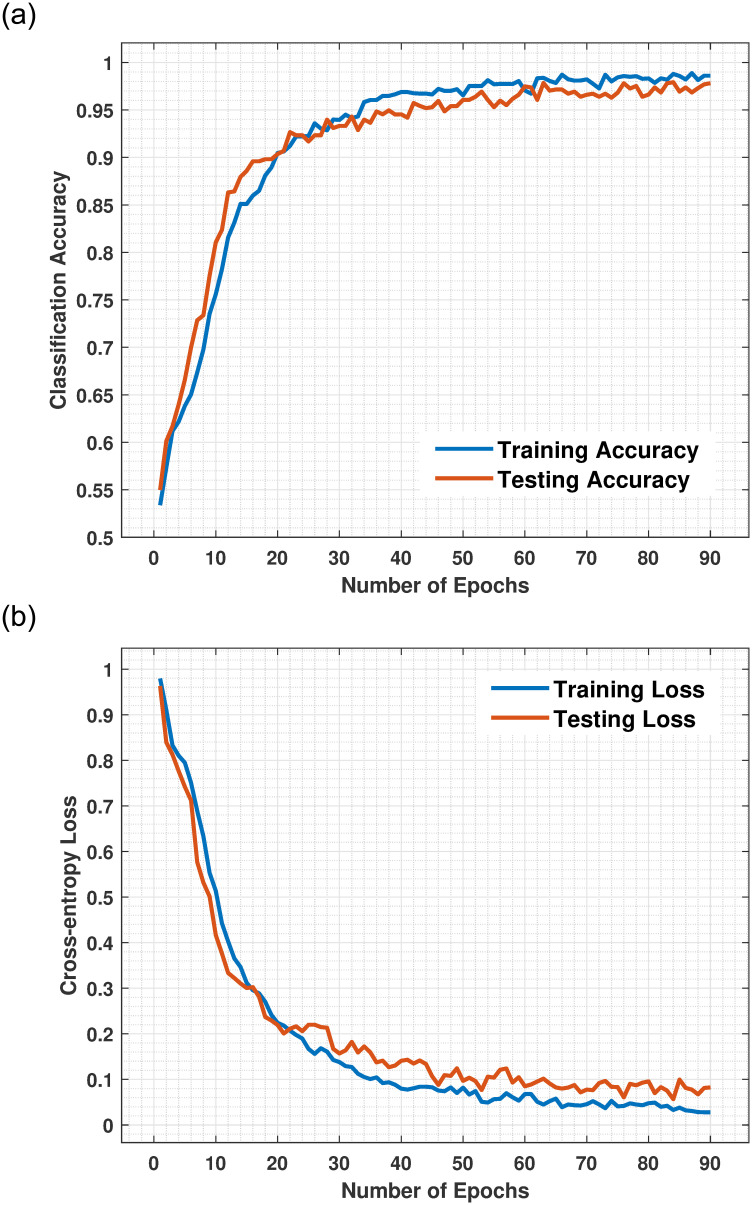
(a) Represent the training and testing accuracy of the proposed model against the training step using a Private dataset. The training accuracy scaled up on each epoch and after the 90^th^ epoch was 0.98 which other models outperform. (b) Represent training and testing cross-entropy loss of the proposed model against the training step using Private dataset. The proposed model generates reduced false-negative results compared to existing DCNN models. The training cross-entropy loss function is declining continuously on every epoch and reduced to a minimum equal to 0.068 at the last epoch.

**Fig 6:**
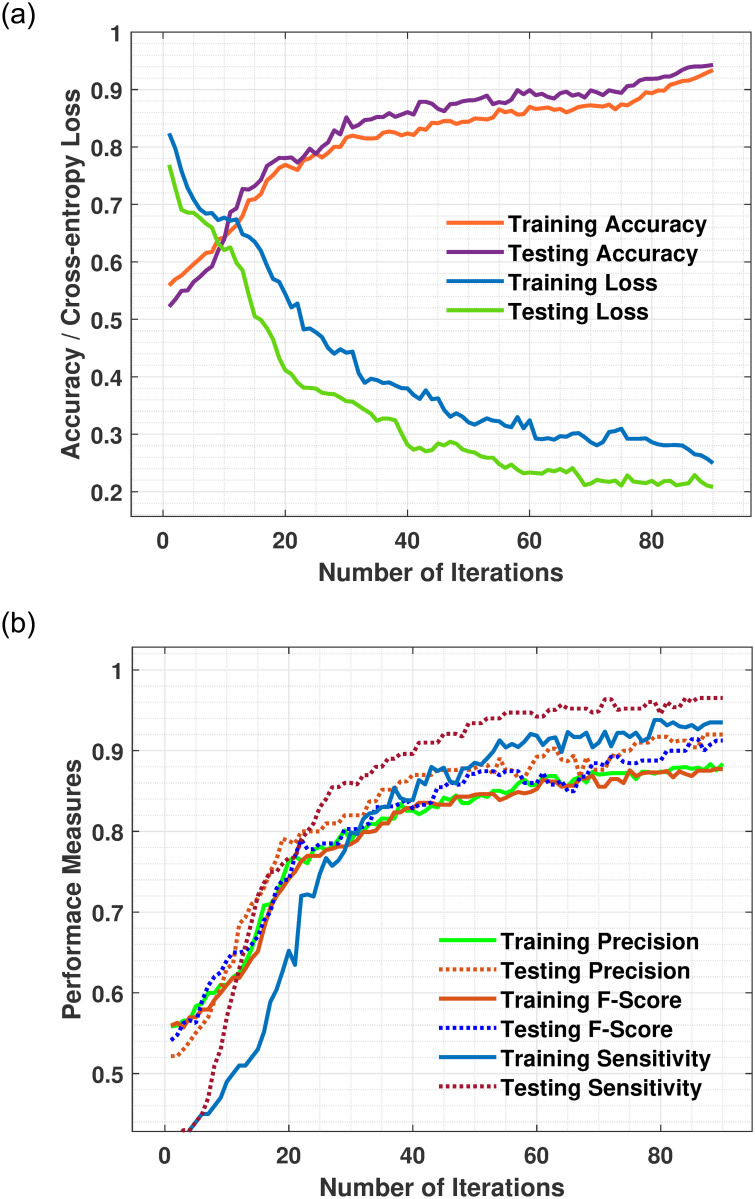
(a) Depicts the suggested system’s accuracy and cross-entropy loss throughout the training phase. The ascending curves indicate the proposed system’s accuracy, while the descending curves indicate the proposed system’s loss value on the training and testing MIAS dataset. The training and testing curves are nearly identical, suggesting that the model was appropriately trained. (b) Training and testing precision, F-score and sensitivity of proposed model using MIAS dataset.

The training and testing curves (accuracy and loss) were plotted after 90 iteration with each iterations with data augmentation using the softmax layer as illustrated in Figs 5a, 5b and 6a and 6b. Our deep learning-based ConvNet model yielded the best performance and attained remarkable 0.98 training accuracy and 0.97 testing accuracy on Private dataset as plotted in Fig 5a. Contrary to this, as shown in Table 3, the VGGNet19, InceptionV3, ResNet152V2, InceptionResNetV2, and EfficientNetB5 architecture yielded accuracies 0.93, 0.82, 0.82, 0.78, and 0.70, respectively. The training accuracy on the Private dataset achieved 0.98 after the 90^th^ epochs, indicating our model was regularized and well fitted. The findings indicate that the proposed model achieved good training accuracy of 0.94 and a testing accuracy 0.93 on MIAS dataset as plotted in Fig 6a, contrary to this, the CNN pre-trained VGGNet19, InceptionV3, ResNet152V2, InceptionResNetV2, and EfficientNetB5 architecture achieved training accuracies 0.72, 0.73, 0.77, 0.73, and 0.65 as depicts in Table 4. According to the statistical analysis of the accuracy parameters, the proposed model achieved 5%, 16%, 16%, 20%, and 28% higher overall accuracy than VGGNet19, InceptionV3, ResNet152V2, InceptionResNetV2, and EfficientNetB5 on the Private dataset. ConvNet has achieved the most significant degree of testing accuracy and the lowest percentage of testing cross-entropy loss. The generalization disparity (accuracy and loss) between training and testing should be minimal to prevent overfitting the model. Numerous facts are noticed in the acquired findings, confirming that the integrated feature map achieves the most significant outcomes and beats the conventional CNN methods.

**Table 4:** Performance comparison of proposed model with pre-trained models on MIAS dataset.

Algorithms	Precision	Sensitivity	F-Score	Accuracy	AUC
VGGNet19	0.72	0.64	0.76	0.72	0.82
InceptionV3	0.74	0.67	0.77	0.73	0.83
ResNet152V2	0.77	0.74	0.74	0.77	0.87
InceptionResNetV2	0.74	0.77	0.78	0.73	0.84
EfficientNetB5	0.65	0.72	0.67	0.65	0.72
Proposed Model	0.88	0.94	0.91	0.94	0.95

The training and testing cross-entropy loss or error rate of the suggested ConvNet method with softmax layer on Private dataset as plotted in Fig 5b and on MIAS dataset Fig 6a. The training loss on Private dataset declining continuously on every epoch and reduced to a minimum equal to 0.068 and testing loss 0.0824 at the last epoch. In comparison, the proposed model attained 0.28 training loss and 0.13 testing loss error rate on MIAS dataset. In Fig 5b, after the 9^th^ epochs, the training loss continuously decreases while the training accuracy remains constant over the iterations, while the loss of other DCNN models are higher which shows our model perfectly fitted on the Private dataset. In Fig 6a, for the MIAS dataset after each epoch, the training loss steadily decreases while the training accuracy remains higher until the last epochs as compared to other DCNN pre-trained approaches. The proposed model outperformed in training loss was less than 0.07.

**Fig 7:**
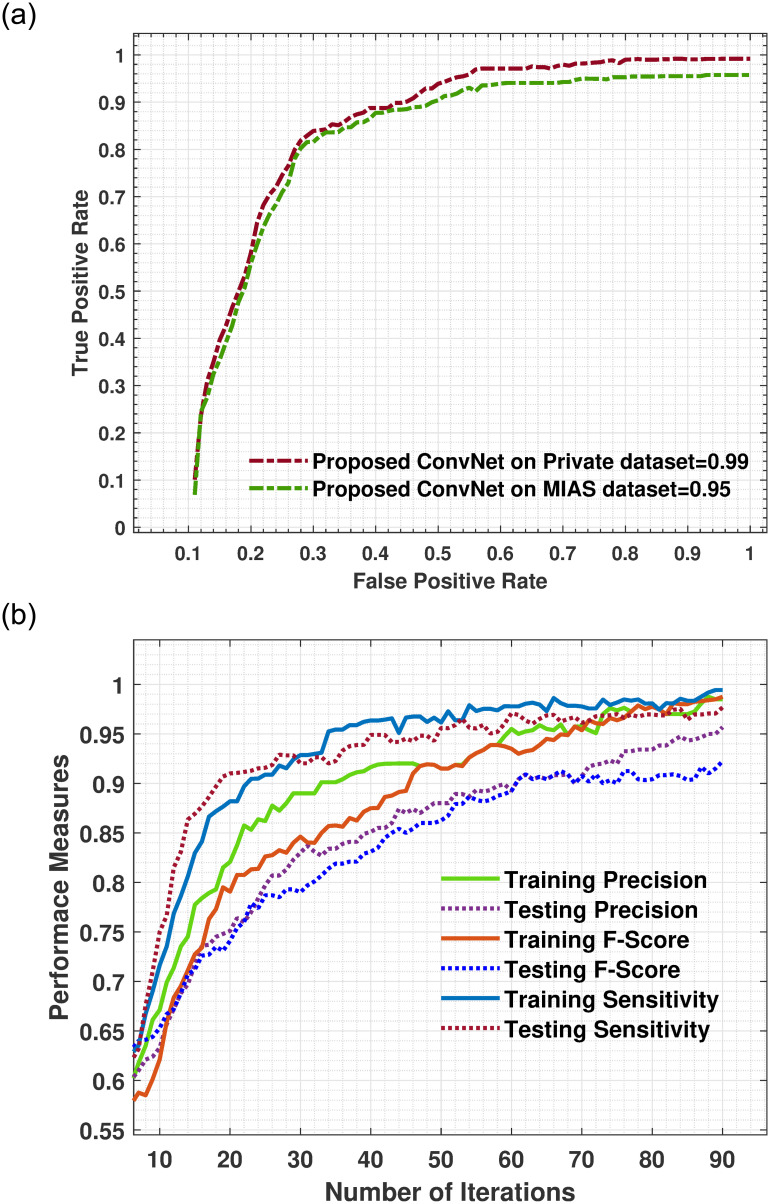
(a) Represent the training and testing area under the curve of the proposed model using Private dataset and MIAS datasets. The graph shows that the proposed model has attained a maximum AUC of 0.99. The experimental findings indicate that the suggested model yields a small number of false positives and high true positives. (b) Training and testing precision, F-score and sensitivity of proposed model using Private dataset. The proposed model has performed well by attaining an excellent sensitivity of 0.99.

The area under the curve (AUC) value evaluated the effectiveness of the models by computing true-positives, false-positives, true-negatives, and false-negatives results used to assess clinical diagnostic systems’ performance. The proposed model achieved a maximum AUC value of 0.99 when tested on the Private dataset and 0.95 on MIAS dataset as shown in Fig 7a, which was higher than the VGGNet19, InceptionV3, ResNet152V2, InceptionResNetV2, and EfficientNetB5 architecture that achieved overall AUC of 0.88, 0.92 0.92, 0.88, and 0.79, respectively. The proposed model obtained promising AUC value and robust performance in reducing false-(positive and negative) rates and in the high number of genuine positives. Additionally, the category-specific ROC curves demonstrate that the ConvNet nearly ultimately differentiates all classes with the highest AUC.

**Fig 8:**
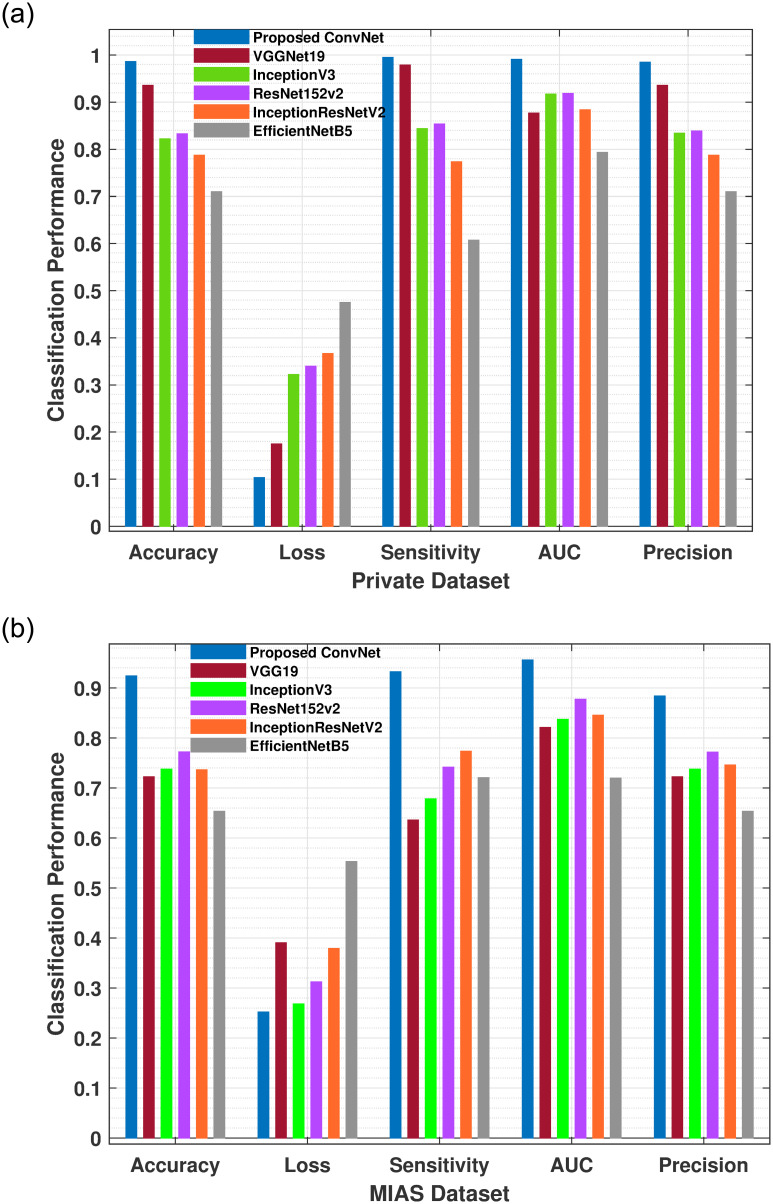
(a) The proposed architecture improves the average accuracy rate, AUC, precision, and sensitivity on Private dataset than MIAS dataset. The proposed model generates reduced false-negative results compared to existing DCNN models. (b) depicts the training accuracy, loss, sensitivity, AUC and precision of proposed ConvNet architecture and DCNN models on MIAS dataset. The proposed model shows promising performance compared to state-of-the-art standard models.

The Figs 6b and 7b, reveals that the proposed method performed excellently and achieved 0.98, 0.98, 0.99 F-score, precision, and sensitivity on the Private dataset and 0.91, 0.88, and 0.94 on the MIAS dataset. In addition, the performance of the F-score, precision and sensitivity of the proposed method was higher than other five pre-trained models as depicts in Tables 3 and 4. Due to the size and blurriness of mammograms, the suggested model performed better on the Private dataset than on the MIAS dataset. In comparison to the current system, our model outperformed it in all classes. Moreover, the overall performance of proposed models on both datasets are illustrated in Fig 8a and 8b. The experimental results demonstrated that a deep convolutional neural network is excellent in classifying ROI of breast masses, which may help physicians and radiologists predict breast cancer earlier.

### Comparison with state-of-the-art existing techniques

The performance of the proposed model has been compared with existing algorithms in terms of accuracy and different performance indicators, as declared in Table 5. It can be observed the proposed model performs excellently due to the data augmentation and transfers learning methods, with an overall accuracy of 0.98. Khan et al. [20] achieve a 0.97 accuracy by employing a CNN model based on transfer learning using 8000 private images. Benzebouchi et al. [33] develop a CNN-based model that outperforms previous methods in classifying breast masses using handmade features with the highest accuracy of 0.97, however the projected outcomes are not very encouraging. Lehman et al. [52] presented a DCNN model for breast mass characterization based on ResNet. The model was validated using 41,479 images classified as dense or non-dense and obtained an accuracy of 0.87. Khan et al. [53] integrated the collected features using the DCNN models VGG, GoogLeNet, and ResNet50, achieving a 0.96 accuracy and a 0.93 AUC. Figs 5a and 6a has been presented the accuracy rate of the current model with a different number of epochs using a softmax-layer. The experimental findings proved that the proposed CNN-based architecture performs betters than conventional systems in accuracy and computation time.

**Table 5:** Comparison of proposed approach with state-of-the-art conventional schemes in terms of accuracy rate. The experimental findings demonstrate the effectiveness of the suggested approach, with an overall training 0.98 accuracy.

Author and (Year)	Method Used	Datasets	Challenges	Aug	ACC
Agnes et al. [21]	MA-CNN	322 MIAS	Automatic mammogram categorization	Yes	0.96
Albalawi et al. [54]	CNN	322 MIAS	Breast mass segmentation	Yes	0.96
Ting et al. [41]	CNNI-BCC	322 MIAS	Breast mass segmentation and grading	Yes	0.90
Chougrad et al. [19]	TL-Based CNN	322 MIAS	ROI segmentation and classification	Yes	0.97
Sha et al. [55]	CNN+SVM	322 MIAS	Segment and classify the breast cancerous areas	Yes	0.92
Tan et al. [27]	CNN	322 MIAS	Classify breast mass as benign or malignant	Yes	0.85
Sannasi et al. [56]	ELM	322 MIAS	Automatic breast lesion diagnoses	Yes	0.91
Duraisamy et al. [57]	DL-CNN	322 MIAS	Mammogram contour segmentation	Yes	0.90
Ragab et al. [26]	CNN+SVM	322 MIAS	Breast mass feature extraction and grading	Yes	0.97
**Proposed Model**	ConvNet	322 MIAS 580 Private	Classification of breast masses intensities	Yes	0.98

## Discussions

Regular mammography screening has become widely known as the most effective method of detecting breast cancer in its earliest stages. However, radiologists’ mammogram-based diagnosis is highly likely to false positives, leading to needless imaging and tumor biopsies. Although the potential of developing deep learning techniques to help in masses screening is intriguing, earlier research has seldom focused on decreasing needless biopsies. Besides sustaining radiologists’ performance in detecting breast masses, a deep learning framework is intended to perform a more decisive role in determining whether lesions are cancerous or non-cancerous. This discrimination is hugely beneficial for suspicious-appearing yet eventually benign results leading to additional biopsies by the radiologist. A few types of breast cancer masses, such as spiculated and ill-defined lesions, still possess accurate detection and classification barriers. The clinical signs of the dense breast are not entirely apparent. However, it is complicated to identify dense lesion features and correctly classify lesions. As a result, the standard CAD systems have fewer challenges in extracting low-level features such as texture and non-textured characteristics to diagnose breast masses.

This study proposed a method for extracting local features from small image patches inside high-resolution mammography. We demonstrated that it is essential to add tiny features confined inside an ROI region to boost the performance of deep learning models for classifying localized masses on high-resolution images. The proposed model can be used for automated annotations to mitigate the annotation cost. Furthermore, the proposed model accurately locates the breast masses during testing and training to avoid redundant tests and reduce the patient call-back rate. Consequently, the presented model performs well in detecting and classifying extremely dense breast masses with distinct shapes, edges, and sizes containing bright normal tissue, which is similar to abnormal masses.

The proposed ConvNet model performs well on the Private and MIAS datasets, with accuracy of 0.98 and 0.94, respectively. Fig 8a and 8b illustrates the overall accuracy, loss, precision, sensitivity and AUC of each proposed models using both datasets. We enhanced the hyperparameters of all five proposed DCNN pre-trained models by adjusting the last layer during training to diagnose breast masses effectively. Detected masses are directly used in the classification stage, which reduces the model’s complexity and computational time. The projected model takes an average testing time and has moderate computational complexity and a quicker processing speed for detecting and classifying breast masses. It may assist further in decreasing needless biopsies by acting as a second reader where radiologists are unsure about the results. As a result, the proposed model has great potential for clinical procedures that provide radiologists with a precise way for quickly computing breast masses and monitoring disease development.

We acknowledge our study suffers from the inherent limitations of observational studies. For instance, we did not interpret the degree of challenges associated with various kinds of breast cancer, which is clinically significant. One of the proposed method’s limitations was the lack in the availability of mammography images data. We will perform this in our future projects.

## Conclusion and future work

Radiologists often have difficulties interpreting patient imaging data correctly, assessing the patient’s health, and detecting benign and malignant masses. Breast cancer mortality in high-risk women is significantly reduced when mammogram interpretation is accurate and leads to effective treatment. This study provides a ConvNet and five DCNN architectures for diagnosing and classifying breast cancer masses, enabling radiologists to detect even the smallest breast masses in their early stages. The transfer learning paradigm is used to enhance the pre-trained DCNN by fine-tuning of hyperparameter. Additionally, the proposed work revealed how image preprocessing and data augmentation strategies may help overcome dataset size bottleneck and mitigate overfitting. We anticipate that the proposed model is very promising and will provide an excellent automated toolkit to heighten prevailing clinical assessment and assist in experts’ decision-making processes. The experimental findings indicate that our method yielded remarkable training accuracy of 0.98, testing accuracy of 0.97, high sensitivity of 0.99, F-Score of 0.98, and AUC of 0.99. Furthermore, as future work, the proposed framework’s efficiency and accuracy can be enhanced by integrating the patches information into the suggested classification algorithm to improve effectiveness and the likelihood of obtaining a correct prediction.

## Abbreviations

BCDR: Breast Cancer Digital Repository
BIRADS: Breast Imaging-Reporting and Data System
CBIS-DDSM: Curated Breast Imaging Subset of DDSM
CNN: Convolutional Neural Network
ConvNet: Convolutional Neural Network
DDSM: Digital Database for Screening Mammography
DICOM: Digital Imaging and Communications in Medicine
GLCM: Gray-level Co-occurrence Matrix
MIAS: Mammographic Image Analysis Society
MRI: Magnetic Resonance Image
PGM: Portable Gray Map
RCNN: Regions with Convolutional Neural Networks
ReLU: Rectified linear units
ResNet: Residual neural network
RF: Random forest
RMS-Prop: Root Mean Square Propagation
ROI: Regions of Interest
RProp: Resilient Propagation
SGD: Stochastic Gradient Descent
SVM: Support Vector Machine
YOLO: You-Only-Look-Once
